# Optimisation, Synthesis, and Characterisation of ZnO Nanoparticles Using *Leonotis ocymifolia* (*L. ocymifolia*) Leaf Extracts for Antibacterial and Photodegradation Applications

**DOI:** 10.3390/ijms252111621

**Published:** 2024-10-29

**Authors:** Dorcas Mutukwa, Raymond Tichaona Taziwa, Shepherd Masimba Tichapondwa, Lindiwe Khotseng

**Affiliations:** 1 Department of Chemistry, University of Western Cape, Robert Sobukwe Road, Private Bag X17, Bellville 7535, South Africa; lkhotseng@uwc.ac.za; 2 Department of Applied Sciences, Faculty of Natural Sciences, Walter Sisulu University, Old King William Town Road, Potsdam Site, East London 5200, South Africa; 3 Department of Chemical Engineering, Sustainable Environmental and Water Utilisation Processes Division, University of Pretoria, Pretoria 0028, South Africa; shepherd.tichapondwa@up.ac.za

**Keywords:** green synthesis, plant-mediated, biomolecules, antibacterial agents, photocatalysts, photocatalysis, dyes, biosynthesis, antimicrobial

## Abstract

This work presents a green synthesis route, which utilises extracts from an indigenous plant in South Africa, eastern and southern Africa that is understudied and underutilised, for preparing zinc oxide nanoparticles (ZnO NPs). This study involved optimisation of the green synthesis method using *Leonotis ocymifolia* (L.O.) extracts and performing comparative studies on the effects of using different zinc (Zn) salt precursors; zinc sulphate heptahydrate (Z001) and zinc acetate dihydrate (Z002) to synthesise the ZnO NPs. The comparative studies also compared the L.O-mediated ZnO NPs and chemical-mediated ZnO NPs (Z003). The as-prepared ZnO NPs were tested for their effectiveness in the photodegradation of methylene blue (MB) dye. Furthermore, antibacterial studies were conducted using the agar well diffusion method on *Escherichia coli* (*E. coli*) and *Staphylococcus aureus* (*S. aureus*) bacteria. The structural, morphological, and optical characteristics of the synthesised ZnO NPs were analysed using XRD, FTIR, SEM, EDS, DRS, and BET techniques. The XRD results indicated that the L.O-mediated ZnO NPs had smaller crystallite sizes (18.24–19.32 nm) than their chemically synthesised counterparts (21.50 nm). FTIR confirmed the presence of biomolecules on the surface of the L.O-mediated NPs, and DRS analysis revealed bandgap energies between 3.07 and 3.18 eV. The EDS results confirmed the chemical composition of the synthesised ZnO NPs, which were made up of Zn and O atoms. Photocatalytic studies demonstrated that the L.O-mediated ZnO NPs (Z001) exhibited a superior degradation efficiency of the MB dye (89.81%) compared to chemically synthesised ZnO NPs (56.13%) under ultraviolet (UV) light for 240 min. Antibacterial tests showed that L.O-mediated ZnO NPs were more effective against *S. aureus* than *E. coli*. The enhanced photocatalytic and antibacterial properties of L.O-mediated ZnO NPs highlight their potential for environmental remediation and antimicrobial applications, thus supporting sustainable development goals.

## 1. Introduction

In recent years, ZnO nanoparticles (NPs) have attracted significant attention among metal oxide NPs owing to their unique optical and chemical properties, which differ from the properties of bulk ZnO [1]. This is due to the high surface-area-to-volume ratio and the influence of quantum effects [2]. These unique properties have allowed the application of ZnO NPs in various fields such as catalysis [3], environmental remediation [4], biomedicine [5], cosmetics, gas sensing, etc.

The green synthesis of ZnO NPs using biological resources, especially plants, has been gaining attention from researchers as it offers a sustainable alternative to chemical and physical methods. This approach is not only simpler and cheaper, but it also eliminates the need for toxic solvents, making it environmentally friendly [6]. Plant extracts from species such as *Passiflora caerulea* [7], *Agathosma betulina* [8], *Hibiscus sabdariffa* [9], *Tilia Tomentosa* [10], *Artocarpus heterophyllus* [11], *Parthenium Hysterophorus* [12], *Calotropis procera* [13], among others, have been successfully employed in the synthesis of ZnO NPs for different applications, including photocatalysis, photovoltaics, biomedicine, and gas sensing.

*Leonotis ocymifolia* (L.O.) is a medicinal plant from the Lamiaceae family that is indigenous to the eastern and southern parts of Africa. Traditionally, it has been used to treat ailments such as hypertension, eczema, anaemia, diabetes, and skin irritations. In South Africa, the plant is widespread in the four northern provinces and the Free State, Western Cape, KwaZulu-Natal, and Eastern Cape provinces [14]. Despite its abundance, only a few studies have explored the L.O. extracts for medicinal applications. Alemu et al. [15] reported the presence of biomolecules such as saponins, tannins, terpenoids, phenolics, alkaloids, and flavonoids in the preliminary phytochemical screening of an 80% methanol extract of L.O. leaves. These biomolecules can facilitate the reduction of Zn salt precursors to ZnO NPs. Additionally, the biomolecules can act as stabilising or capping agents during the synthesis of NPs, thus eliminating the need for the toxic chemicals commonly used in conventional chemical methods [16]. Therefore, this work will report, for the first time, the use of L.O. leaf extracts to prepare ZnO NPs, thus utilising an abundant African plant resource that is understudied and underexplored.

The wastewater from industries such as textile, food, pharmaceutical, paint, plastic, and cosmetics are some of the contributors to organic dyes in waterbodies. The presence of these organic dyes in the environment is very detrimental to the environment, aquatic ecosystems, and human health [17,18]. Hence, removing these pollutants from the wastewater is crucial before its discharge into waterbodies. In recent years, the photocatalytic degradation of dyes using semiconductor NPs has been receiving tremendous attention as an alternative to conventional wastewater treatment methods. This is because these conventional methods are limited by their inability to fully degrade dyes due to the high solubility and non-biodegradable nature of the dyes [19]. ZnO NPs have emerged as a promising material for photocatalytic dye degradation due to their strong oxidising ability, low toxicity, low cost, and high photostability [20].

In addition to their photocatalytic properties, ZnO NPs have demonstrated significant potential for antibacterial applications. For decades, antibiotics have been the primary therapeutic agents for combating bacterial infections. Since their introduction in the healthcare system, the use of antibiotics has led to a large reduction in the bacterial infectious disease mortality rate. However, the over-reliance on antibiotics has led to the emergence of antibiotic-resistant bacteria, which has become a global health threat. As a result, the development of alternative antibacterial agents that are safe and cheap is crucial to help fight antibiotic resistance. Green synthesised ZnO nanoparticles have emerged as a promising alternative to traditional antibiotics, showing encouraging results in the fight against antibiotic resistance [21]. This study, therefore, also aims to investigate the antimicrobial efficacy of L.O-mediated ZnO NPs against *Staphylococcus aureus* (*S. aureus*) and *Escherichia coli* (*E. coli*), highlighting their potential as an effective antibacterial agent.

## 2. Results and Discussion

### 2.1. Qualitative Analysis of L.O. Extracts

Table 1 shows the preliminary screening of biomolecules that were present in the L.O. leaf extracts. The results indicate the presence of various biomolecules, such as phenols, flavonoids, and terpenoids. Biomolecules from plant extracts have been reported to serve two major functions: (1) reducing agents, and (2) stabilising agents. They play a role in the reduction of metal salts to NPs and the stabilisation of the NPs, thereby controlling their physical and chemical properties such as particle size, shape, and crystallinity. This inherently affects their use for various applications [22].

### 2.2. Optimisation of Synthesis Parameters

**Figure 1:**
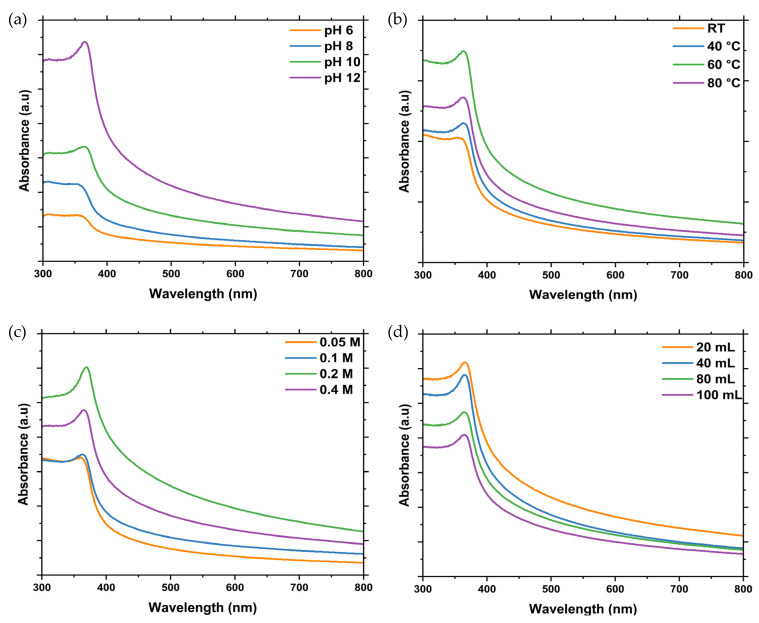
The UV-Vis spectra of the L.O-mediated ZnO NPs synthesised at different (**a**) pH, (**b**) reaction temperature, (**c**) Zn salt concentration, and (**d**) plant extract volume. RT = room temperature.

Reaction parameters such as the pH, reaction temperature, concentration of Zn salt precursor, annealing temperature, volume of extract, and reaction time are crucial during the plant-mediated synthesis of ZnO NPs. These parameters significantly influence the properties of the synthesised ZnO NPs such as particle size, shape, and yield. To achieve high-quality ZnO NPs with optimal characteristics for dye photodegradation and antibacterial activity, this study systematically optimised these parameters. The optimisation process was monitored using ultraviolet-visible spectroscopy (UV-Vis). Figure 1a–d shows the results of the optimisation of the various reaction parameters.

The optimisation of the pH was studied by increasing the pH of the reaction mixture from 6 to 12 using 2 M sodium hydroxide while other reaction parameters remained constant. Absorption peaks were observed for all the pHs, with the peak intensity increasing with an increase in pH, as shown in Figure 1a. The increase in peak intensity correlated with the reduction of zinc acetate dihydrate to ZnO NPs, where the characteristic absorption peak arose from the transition of electrons from the valence band (VB) to the conduction band (CB) [23]. The highest absorbance intensity was observed at a pH of 12, which was therefore identified as the optimum pH for ZnO NP synthesis using L.O. extracts and is consistent with observations for ZnO NPs synthesised using *Grewia asiatica* [24]. From Figure 1b, it can be observed that absorption peaks were present for all temperatures. However, the absorbance intensity increased with an increase in temperature from RT to 60 °C. Further increase in temperature to 80 °C resulted in a decrease in absorbance intensity. Hence, 60 °C was found to be the optimum temperature for synthesising ZnO NPs using L.O. extracts. The reduction in absorbance intensity at 80 °C can be attributed to the instability of the L.O. biomolecules at high temperatures, resulting in a decrease in their reducing power. A similar temperature was utilised for the synthesis of ZnO NPs using *Morus nigra* [25]. No significant shifts in absorption peaks were observed during the pH and temperature optimisations.

The optimisation of the Zn salt concentration was studied by increasing the concentration of the zinc acetate dihydrate from 0.05 to 0.4 M. The results from Figure 1c reveal that the absorbance intensity increased from 0.05 to 0.2 M and then decreased when the concentration was increased to 0.4 M, indicating that 0.2 M was the optimal concentration for ZnO NPs synthesis. Increasing the concentration of Zn salt precursor from 0.05 to 0.2 M increased the concentration of Zn ions available for reduction by the L.O. extracts, which resulted in an increased yield and hence increased absorption intensity. However, a further increase in the Zn precursor concentration beyond 0.2 M resulted in a decrease in the absorption intensity due to saturation of the L.O. biomolecules. A similar concentration of Zn salt precursor was successfully utilised to synthesise ZnO NPs using *Achillea nobilis* extract [26]. The volume of plant extract was optimised by changing the plant volume from 20 to 100 mL. The absorbance intensity decreased with an increase in volume from 20 to 100 mL. This was due to the presence of excess biomolecules from the L.O. extracts when the volume went beyond 20 mL, which affected the nucleation process, thus leading to agglomeration and hence a lower absorption intensity. From the results shown in Figure 1d, 20 mL was considered the optimum volume for synthesising ZnO NPs using L.O. leaf extracts.

### 2.3. X-Ray Diffraction (XRD) Analysis

**Figure 2:**
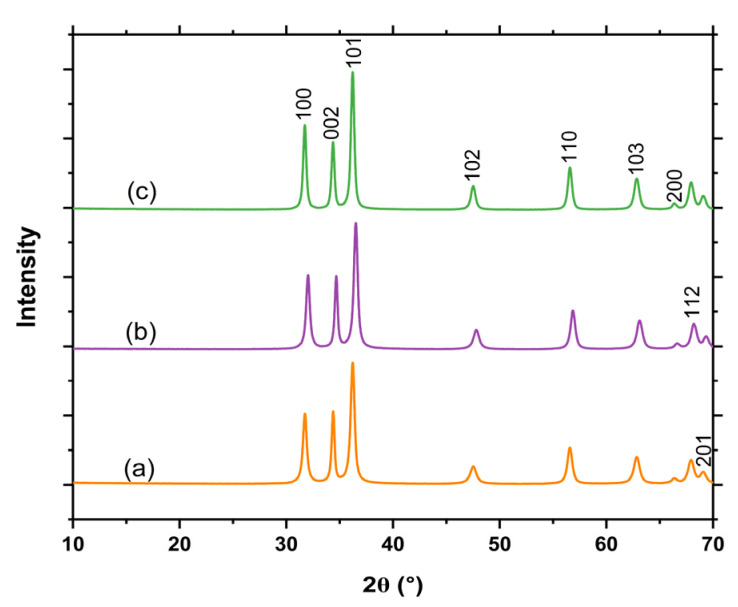
XRD peaks of the ZnO NPs: (**a**) Z001, (**b**) Z002, and (**c**) Z003.

XRD was carried out to investigate the crystalline structure of the L.O-mediated ZnO NPs (Z001 and Z002) and ZnO NPs synthesised without the L.O. extracts (Z003), as shown in Figure 2. All the peaks from the as-synthesised ZnO NPs were consistent with the hexagonal wurtzite phase of ZnO, as indexed by the Joint Committee on Powder Diffraction Standards (JCPDS) Card Number: 36-1451. The sharp and intense diffraction peaks confirmed that the ZnO NPs were well crystallised. No other diffraction peaks from impurities were observed, which confirmed that L.O. extracts could be utilised in the production of high-quality ZnO NPs. Additionally, the average crystallite sizes (D) were calculated using the Debye–Scherrer equation as shown in Equation (1).

(1) $$D=\frac{K\lambda }{\beta cos\theta },$$

where *K* is the Scherrer constant (0.9), λ is the wavelength, β is the full width at half maximum, and *θ* is the Bragg’s angle.

The calculated average crystallite sizes were 18.2, 19.3, and 21.5 nm for Z001, Z002, and Z003, respectively. The Z003 had a larger crystallite size than Z001 and Z002, and this may be attributed to the presence of biomolecules such as alkaloids, phenols, saponins, etc. in the L.O. extracts, which can act as stabilisers, thereby controlling the growth of the nanoparticles. These results are similar to the observations from the XRD calculations made by Ashraf et al. [27], who found that the ZnO NPs synthesised without plant extract (32 nm) had a larger crystallite size than the *Boerhavia diffusa linn*-mediated ZnO NPs (23 nm).

### 2.4. UV-Vis-Diffuse Reflectance Spectroscopy (DRS)

**Figure 3:**
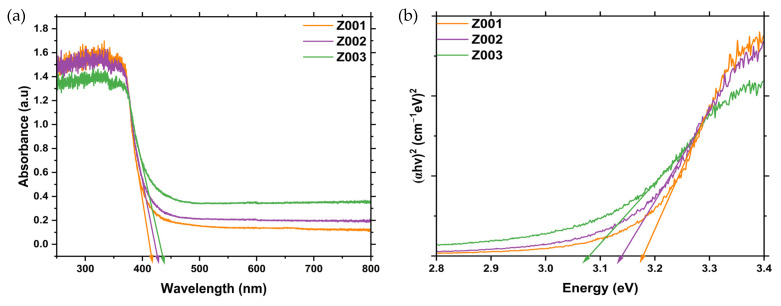
(**a**) DRS spectra of the synthesised ZnO NPs, and (**b**) the Tauc plots of the synthesised ZnO NPs.

The bandgaps of the synthesised ZnO NPs were determined using the data obtained from the DRS analysis, and the spectra are given in Figure 3a. The optical bandgaps were estimated using the Tauc equation given in Equation (2) below.

(2) $$\alpha hv=A{\left(hv-E_{g}\right)}^{2},$$

where *α* is the absorption coefficient, *hv* is photon energy, *A* is the band tailing constant, and *E_g_* is the optical bandgap.

The optical bandgaps were estimated by plotting the graph of (*αhv*)^2^ vs. the *hv* of the synthesised ZnO NPs, as shown in Figure 3b. The estimated absorption edges and the optical bandgaps of the synthesised ZnO NPs are given in Table 2.

The bandgaps of Z001, Z002, and Z003 decreased with an increase in crystallite sizes, as observed from the XRD results. This can be attributed to the increase in the number of atoms as the crystallite size increased, leading to an increased overlapping of orbitals and a narrowing of the width of the bands. This results in a decrease in the energy gap between the VB and CB. Geetha et al. [28] also reported a decrease in bandgap with an increase in crystallite sizes of *Euphorbia jatropa* latex-mediated ZnO NPs.

### 2.5. Fourier Transform Infrared Spectroscopy (FTIR)

**Figure 4:**
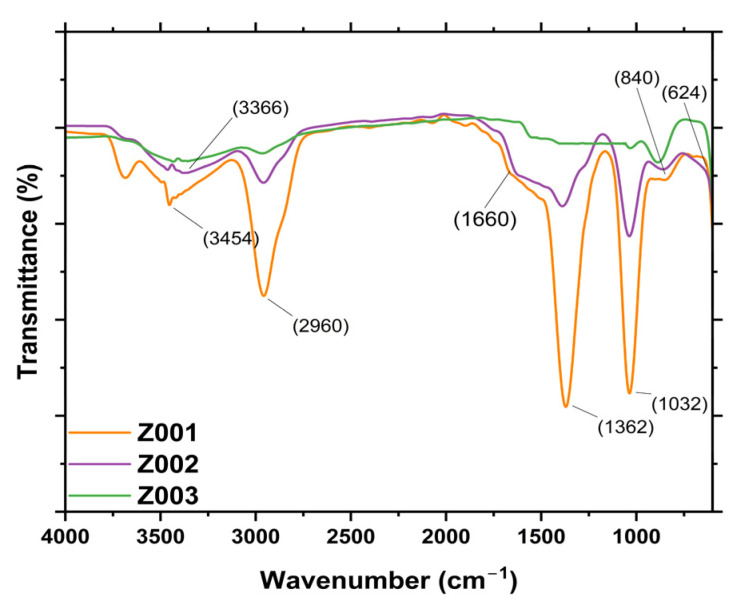
FTIR spectra of the synthesised ZnO NPs.

The FTIR analysis was performed to determine the functional groups on the surface of the synthesised ZnO NPs and to confirm the synthesis of ZnO NPs. According to the literature, Zn−O stretch vibrations, which confirm the synthesis of ZnO NPs, can be observed in the region between 400 and 800 cm^−1^ [29]. The FTIR spectra of the synthesised ZnO NPs are given in Figure 4. In this work, the Zn−O bond was observed around 634 cm^−1^, whereas, in Z002 and Z003, it was observed around 694 cm^−1^. This confirmed the successful synthesis of ZnO NPs using L.O. extracts and the chemical route, thus supporting the XRD results, which confirmed the presence of ZnO NPs. The peaks at around 1034 and 1364 cm^−1^ can be assigned to the C−N stretching of the aliphatic amines and the C−H bend vibration of the alkenes [30]. The peak at 1660 cm^−1^ can be assigned to the C=C stretching and C=O stretching of the carbonyl of the amides and carboxylic acids [31,32]. These functional groups can be attributed to biomolecules such as flavonoids, phenols, and terpenoids. Additionally, the presence of these biomolecules was also confirmed by the qualitative analysis of the L.O. extracts. Therefore, these findings suggest that some biomolecules from the L.O. extracts could have been involved in the reduction and capping of the synthesised ZnO NPs. The peaks around 1660 and 2952 cm^−1^ were also present in the spectrum of Z003 but at very low intensity, and they could be from the zinc acetate ions since zinc acetate dihydrate was the Zn salt precursor used to synthesise Z003 [33].

### 2.6. Morphological and Elemental Analysis

**Figure 5:**
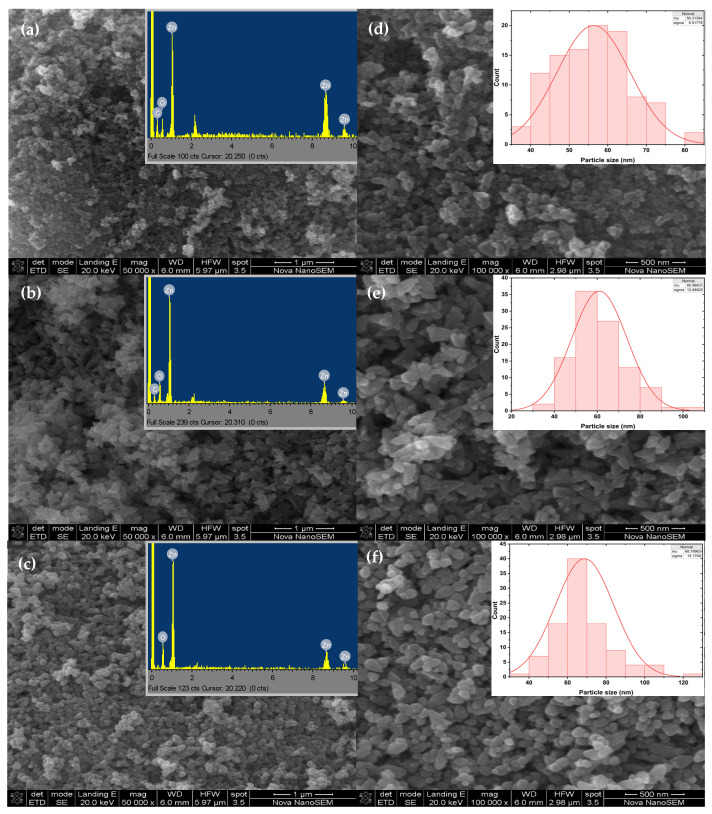
SEM images of the synthesised ZnO NPs at low magnification: (**a**) Z001, (**b**) Z002, and (**c**) Z003. SEM images of the synthesised ZnO NPs at high magnification: (**d**) Z001, (**e**) Z002, and (**f**) Z003. The EDS spectra of the synthesised ZnO NPs in insets: (**a**) Z001, (**b**) Z002, and (**c**) Z003. The particle size distribution graphs in insets: (**d**) Z001 (**e**) Z002, and (**f**) Z003.

The size and shape of the synthesised ZnO NPs were evaluated using scanning electron microscopy (SEM), and the SEM images are depicted in Figure 5. ZnO NPs are well known for exhibiting a variety of morphologies such as rods, wires, hexagons, spherical, and flowers. Gatou and co-workers [34] observed different morphologies from SEM analysis as a result of using different Zn salt precursors to prepare chemical-mediated ZnO NPs. The SEM images revealed flake-shaped particles for the zinc chloride, zinc nitrate, and zinc sulphate, whereas the zinc acetate-mediated ZnO NPs were both spherical and hexagonal. Sekar et al. [35] also reported different ZnO NPs morphologies from the SEM analysis of *Anisomeles malabarica* (*A. malabarica*)-mediated ZnO NPs. The *A. malabarica*-mediated ZnO NPs from the zinc acetate dihydrate were spherical and accumulated into bullets. In contrast, the particles from zinc nitrate were round and accumulated into flower-like shapes. In this study, the Z001 L.O-mediated ZnO NPs were semi-spherical in shape with some agglomeration (Figure 5a,d), whereas the Z002 NPs were quasi-spherical with triangular-like shapes (Figure 5b,e) and the Z003 NPs were semi-spherical (Figure 5c,f). ZnO NPs. These findings are in agreement with reports on the influence of the type of Zn salt precursor on the morphology of ZnO NPs.

The average particle sizes and size distribution were estimated using Image Processing and Analysis in Java (Image J) Java 8 software, with the particle distribution graphs given in insets d–f in Figure 5. The average particle sizes were estimated to be 56.3, 61.0, and 68.8 nm. Z003 had a higher average particle size and size distribution than Z001 and Z003. The particle size distributions were 37.6–81.4, 30.7–107.1, and 38.8–127.0 nm for Z001, Z002, and Z003, respectively, with normal distributions. Muthuvel et al. [36] also reported chemical-mediated ZnO NPs with higher particle sizes than *Solanum nigrum*-mediated ZnO NPs, with particle sizes of 72 and 49 nm, respectively, from the dynamic light scattering (DLS) analysis. The smaller particle size of L.O-mediated compared to chemical-mediated ZnO NPs can be attributed to the capping of the ZnO NPs by the L.O. biomolecules leading to better size control. Additionally, the slight agglomeration of the L.O-mediated ZnO NPs can be attributed to the high surface energy and the chemical makeup of the biomolecules on the surface of the NPs [37].

The elemental analysis using energy dispersive X-ray spectroscopy (EDS) revealed a single peak of zinc and oxygen between about 0.5 and 1 keV and two small peaks of zinc between 8 and 10 keV, as shown in insets a–c in Figure 5 for Z001, Z002, and Z003, respectively. This is similar to the EDS results reported in a study that prepared ZnO NPs using *M. acuminata* and a chemical route [38]. In our work, the EDS spectra of Z001 and Z002 showed the presence of some carbon, whereas no carbon was observed in the spectrum of Z003. Carbon could have been introduced into the L.O-mediated ZnO NPs from the organic compounds present in the L.O. extracts, which are responsible for capping the synthesised ZnO NPs. These findings are also in good agreement with the EDS results reported for *Dolichos Lablab*-mediated ZnO NPs and *Mussaenda frondose*-mediated ZnO NPs, which showed the presence of carbon from the EDS spectrum [39,40].

### 2.7. Brunauer, Emmett and Teller (BET) Analysis

**Figure 6:**
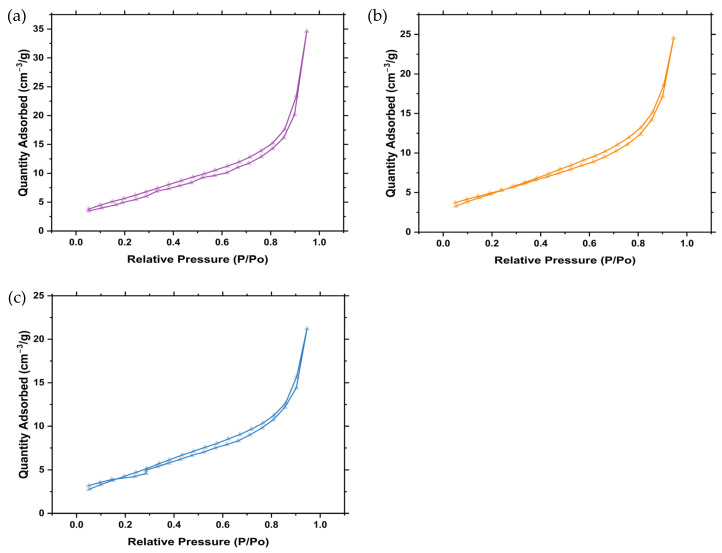
N_2_ adsorption–desorption isotherms of the synthesised ZnO NPs: (**a**) Z001, (**b**) Z002, and (**c**) Z003.

BET analysis was conducted to evaluate the surface area and pore volume of the synthesised ZnO NPs. Analysis of the surface area is important in both antibacterial and photocatalysis studies as it evaluates the active sites that are present for the adsorption of dye molecules, interaction with bacteria, and the generation of reactive oxygen species (ROS) such as hydroxyl radicals and superoxides. The surface area of the synthesised ZnO NPs was determined using BET surface area analysis. The isotherms are given in Figure 6a–c, and it can be observed that the synthesised ZnO NPs followed the Type IV isotherm with a deceleration loop in the low-pressure area (P/Po). The BET surface area, pore volume, and pore diameter are shown in Table 3. Z001 had the highest surface area (20.3 m^2^g) compared to Z002 and Z003 (18.0 and 15.7 m^2^g, respectively). This can be attributed to the smaller particle size of Z001 compared to Z002 and Z003, which was confirmed by the SEM analysis. All the synthesised ZnO NPs were mesoporous because their pore diameter was within the 2–50 nm range.

### 2.8. Photocatalytic Activity of the Synthesised ZnO NPs

**Figure 7:**
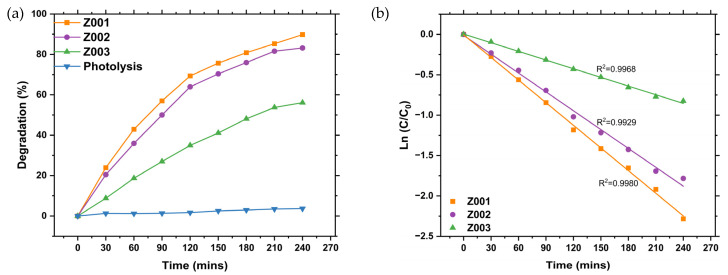
(**a**) Comparative degradation of the MB dye using L.O-mediated ZnO NPs, ZnO NPs without L.O. extracts, and MB dye without a photocatalyst. (**b**) Kinetics study of the degradation of MB dye using L.O-mediated ZnO NPs and ZnO NPs without the L.O. extracts.

The degradation efficiencies of the MB dye under UV exposure for 240 min using Z001, Z002, and Z003 were calculated as 89.81, 83.19 and 56.13%, respectively. The control without the synthesised ZnO NPs photocatalysts revealed negligible photolysis (Figure 7a). Z001 had the highest degradation efficiency compared to Z002 and Z003, as seen in Figure 7a. This is similar to the observations by Kumar et al. [41] in their investigation of the photodegradation of MB dye using *Ruta Chalepensis* (*R. chalepensis*)-mediated ZnO NPs and chemical-mediated ZnO NPs under UV irradiation for 60 min. The *R. chalepensis*-mediated ZnO NPs exhibited a significantly higher degradation efficiency (74.14%) than chemical-mediated ZnO NPs (56.50%).

Despite the smaller bandgap of Z003 compared to Z001 and Z003, it exhibited the lowest degradation efficiency. This could be attributed to the smaller particle size of Z001 than Z002 and Z003, as seen from the SEM results, which resulted in a higher specific surface area, as observed from the BET results. Smaller particles result in a higher surface-to-volume ratio, which provides a higher surface area for the adsorption of dye molecules and the generation of charge carriers responsible for the generation of ROS. Additionally, the better photoactivity of Z001 and Z002 than Z003 can be due to the presence of the biomolecules on the surface of the L.O-mediated ZnO NPs, which may act as photosensitisers resulting in less energy needed for the generation of charge carriers [42]. This resulted in improved degradation efficiency as more ROS were generated, which were responsible for the degradation of the MB dye.

The kinetics studies of the degradation of MB dye using Z001, Z002, and Z003 were performed using the pseudo-first-order model, which can be described by Equation (3). Photodegradation typically follows the Langmuir–Hinshelwood isotherm model.

(3) $$In\frac{C}{C_{0}}=-kt,$$

where *C* is the MB dye at time *t*, *C*_0_ is the MB dye concentration at *t* = 0, *k* is the velocity constant of a pseudo-first-order, and *t* is the time in minutes.

The photodegradation of MB dye using Z001, Z002, and Z003 followed the pseudo-first-order model, as shown in Figure 7b. The R^2^ values were 0.9980, 0.9929, and 0.9968 for Z001, Z002, and Z003, respectively, which were an excellent fit to the pseudo-first-order model. The rate constants from the graph plot of the natural logarithm of (*C*/*C*_0_) vs. time were 9.35 × 10^−3^, 7.73 × 10^−3^, and 3.58 × 10^−3^ min^−1^ for Z001, Z002, and Z003, respectively. Z001 had the highest rate constant, followed by Z002, compared to Z003. Z001 showed better photoactivity against MB dye than Z002 and Z003; therefore, it was used in the optimisation experiments for the photodegradation of MB dye using L.O-mediated ZnO NPs. 

#### 2.8.1. Optimisation of the Photodegradation of MB Dye Using L.O-Mediated ZnO NPs

**Figure 8:**
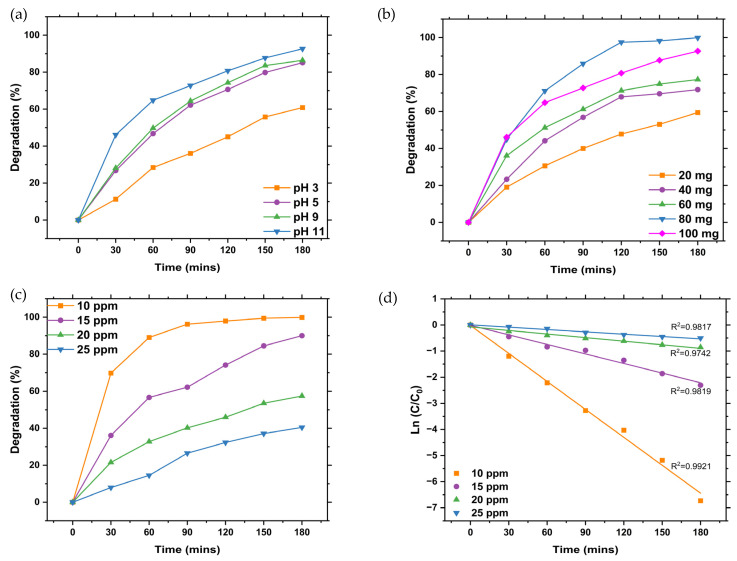
Graphs of the degradation efficiencies of MB dye using L.O-mediated at different (**a**) pHs, (**b**) dosages, and (**c**) initial MB dye concentration. (**d**) Kinetics study of the degradation of MB dye using L.O-mediated ZnO NPs.

The pH is a critical factor in the photodegradation process as it influences the surface chemistry, solubility, and adsorption characteristics of photocatalysts. To evaluate the effect of the pH on the photodegradation of MB dye using Z001, the pH of the suspension was varied from 3 to 11 while keeping other parameters constant and exposing the mixture to UV light for 180 min. Figure 8a demonstrates that the degradation efficiency of MB dye with Z001 is pH-dependent, increasing with rising pH levels. The highest degradation efficiency was achieved at a pH of 11, establishing it as the optimum pH for MB dye degradation when using L.O-mediated ZnO NPs. This finding aligns with similar studies on ZnO NPs by Kazeminezhad and Sadollahkhani [43].

The point of zero charge (pH_pzc_) of ZnO is 9 [44,45], and at pH > pH_pzc_ (pH 11), the surface of ZnO is negatively charged, whereas MB dye is a cationic dye and, therefore, MB dye molecules are positively charged. Hence, the MB dye molecules enhanced the adsorption on the ZnO NPs surface due to the strong electrostatic attraction between the cationic dye molecules and the negatively charged ZnO NPs. However, at pH < pH_pzc_ (pH 3 and 5), the surface of the ZnO NPs and MB dye molecules were both positively charged. This resulted in a repulsion between the ZnO NPs and the dye molecules, leading to reduced adsorption of the MB dye molecules on the surface of the NPs. This led to a reduced degradation efficiency as adsorption is an important step in the photodegradation of dyes [46].

The influence of the catalyst dosage on the photodegradation of MB dye was investigated while other parameters remained constant. Figure 8b shows that the degradation efficiency increased with an increase in the L.O-mediated ZnO NP dosage from 20 to 80 mg and decreased when the dosage was increased to 100 mg. Therefore, an 80 mg dosage was the optimum dosage for the degradation of MB dye using L.O-mediated ZnO NPs. The increase in degradation efficiency as the L.O-mediated ZnO NPs dosage increased can be attributed to an increase in the available active sites for the adsorption of the MB dye molecules and generation of ROS that take part in the degradation of the MB dye molecules, which leads to increased activity. However, when the optimum dosage has been reached, a further increase in the dosage increases the turbidity of the suspension, which results in increased light scattering. This leads to reduced charge carrier generation and limits the generation of ROS, thus reducing the efficiency [47].

The influence of initial dye concentration was studied to determine the optimum concentration required for the efficient degradation of MB dye using L.O-mediated ZnO NPs while other parameters remained constant. The degradation efficiency decreased with an increase in the initial dye concentration, as shown in Figure 8c. The degradation efficiency decreased from about 99 to 40% when the initial dye concentration was increased from 10 to 25 ppm, thus making 10 ppm the optimum concentration for degradation of the MB dye using L.O-mediated ZnO NPs. The reduction in degradation efficiency as the initial dye concentration increased was due to the reduction in the light penetration through the dye solution. This led to a reduction in the ROS generated, thus resulting in reduced degradation efficiency.

The kinetics studies of the degradation of the MB dye using L.O-mediated ZnO NPs at different initial dye concentrations were performed using the pseudo-first-order model. As seen in Figure 8d, the degradation of MB dye at different initial dye concentrations using L.O-mediated ZnO NPs followed the pseudo-first-order model with R^2^ values of 0.9921, 0.9819, 0.9742, and 0.9817 for 10, 15, 20, and 25 ppm of the initial MB dye concentration, respectively. The rate constants were 3.568 × 10^−2^, 1.22 × 10^−2^, 4.56 × 10^−3^, and 2.99 × 10^−3^ min^−1^ for 10, 15, 20, and 25 ppm of the initial MB dye concentration, respectively.

#### 2.8.2. Reusability

**Figure 9:**
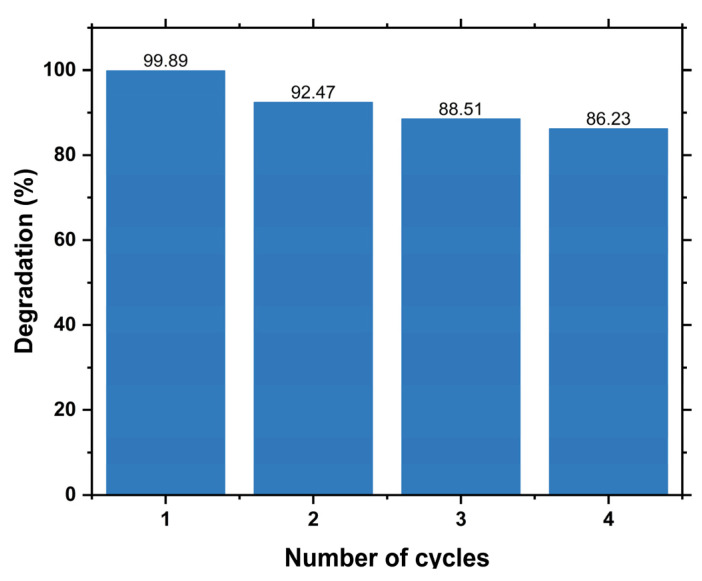
Degradation efficiency of the MB dye using L.O-mediated NPs after four cycles.

The reusability of the L.O-mediated ZnO NPs for the photodegradation of MB dye was conducted at a pH of 11, an 80 mg dosage, and 10 ppm of initial dye concentration. The catalyst was recovered after every cycle by centrifuging, washing several times with distilled water and drying in the oven. The recovered catalyst was then reused for the photodegradation experiments. The degradation efficiencies of MB dye using L.O-mediated ZnO NPs after four photodegradation cycles are shown in Figure 9. The degradation efficiency decreased from 99.89 to 86.23% after four cycles. The degradation efficiency decreased by about 13%, possibly due to the loss of photocatalyst during the washing process and due to the blockage of the active sites by the degradation products of the MB dye [48]. The degradation products limit the light absorption and adsorption of MB dye molecules on the surface of the L.O-mediated ZnO NPs, thereby reducing the degradation efficiencies. These results show the potential of L.O-mediated ZnO NPs as a photocatalyst in the photodegradation of dyes from wastewater. Fu and Fu [49] reported similar stability to the *Plectranthus amboinicus*-mediated ZnO NPs in the photodegradation of methyl red dye. The degradation efficiency decreased from about 92 to 85% in four degradation cycles under UV irradiation in 180 min. The stability of the degradation efficiency of the synthesised L.O-mediated ZnO NPs can be enhanced by immobilising the NPs on supports such as zeolites, polymers, glass substrates, and hydrogels, as well as by compositing them with magnetic materials. These measures can improve the ease of recovery of the nanocatalyst from the dye/catalyst matrix, thereby reducing the loss of catalyst as a result of washing and centrifuging the dispersed NPs in the dye/catalyst matrix.

### 2.9. Antibacterial Activity

The antibacterial activity of the synthesised ZnO NPs was tested against gram-negative (G−) *E. coli* and gram-positive (G+) *S. aureus*. The ZOI of the synthesised ZnO NPs, positive control (ciprofloxacin), and negative control against *S. aureus* are given in Table 4.

**Figure 10:**
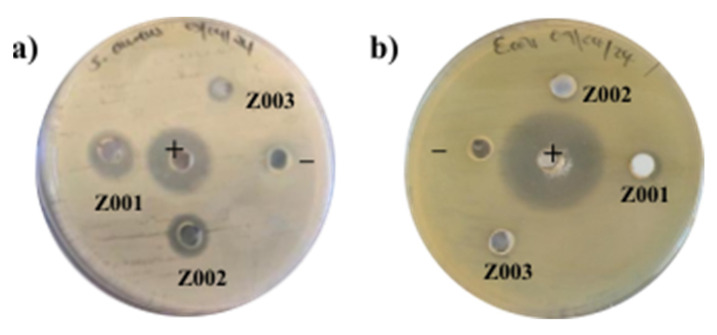
ZOI of the synthesised ZnO NPs, positive control, and negative control against (**a**) *S. aureus* and (**b**) *E. coli*.

Z001 and Z002 exhibited better antibacterial activity on *S. aureus* than Z003, as can be seen in Figure 10a. The difference in the antibacterial activity of the L.O-mediated ZnO NPs and chemical-mediated ZnO NPs may be due to the synergic efforts of the ZnO NPs and the biomolecules from the L.O. extracts on the surfaces of Z001 and Z002. Additionally, the smaller particle size and higher specific surface area of Z001 compared to Z003 may have resulted in a difference in the antibacterial activity. This is because smaller particles can penetrate bacteria cell membranes where they cause damage to the bacteria. Moreover, the higher surface-to-volume ratio of the smaller particles resulted in the enhanced generation of ROS, which disrupted the bacteria’s activities, leading to the death of the bacteria [50]. All the synthesised ZnO NPs exhibited negligible antibacterial efficacy on the G− *E. coli*, as seen in Figure 10b. Inamdar et al. [51] also reported stronger antibacterial activity against G+ than G−, as supported by the higher minimum inhibition concentration for *E. coli* (25 µg/mL) compared to *S. aureus* (0.00025 µg/mL) using *Mimosa pudica*-mediated ZnO NPs. This may be due to the presence of a lipid bilayer in the G− bacteria that is absent in G+ bacteria and may be harder to penetrate [52].

## 3. Materials and Methods

The L.O. leaves were collected from Cefani Nursery, Cintsa West, East London, Eastern Cape, South Africa. To ensure the removal of dust and other foreign materials, the leaves were washed twice, first with tap water and then with distilled water. The washed leaves were air-dried for four days to eliminate moisture, after which they were ground into a fine powder using an electric blender.

### 3.1. Preparation of Extract

The biomolecules were extracted by placing 30 g of L.O. powder into 600 mL of distilled water and heating the mixture to 80 °C for 1 h. The mixture was filtered using vacuum filtration. The resulting L.O. extracts were then stored in the refrigerator at 4 °C until further use.

### 3.2. Biomolecules Analysis

The L.O. extracts were analysed for the presence of various biomolecules such as flavonoids, alkaloids, tannins, saponins, phenols, glycosides, terpenoids, and proteins using a lead acetate test, Mayer’s test, gelatine test, froth test, ferric chloride test, Killer Killian’s test, Salkowski’s test, and Biuret’s test, respectively, following the procedure reported by Umamaheswari et al. [53] and the methods given below.

Test for flavonoids: The flavonoids were tested using lead (II) acetate by adding a few drops of lead (II) acetate (ACS reagent-Merck, ≥99%) to 2 mL of the extract. The formation of a white-coloured precipitate indicates the presence of alkaloids.

Test for alkaloids: Mayer’s reagent was used to test for alkaloids by adding a few drops to 1 mL of the extract. The Mayer’s reagent was prepared by adding 1.36 g of mercuric chloride (ACS reagent-Merck, ≥99.5%) and 5.00 g of potassium iodide (ACS reagent-Merck, ≥99.0%) to 100 mL of distilled water. The appearance of a cream-coloured precipitate indicates the presence of alkaloids.

Test for tannins: The gelatine test was used to test for tannins and involved adding a few drops of 1% gelatine containing a few drops of 10% sodium chloride (ACS reagent-Merck, ≥99.0%). The appearance of a white-buff colour indicates the presence of tannins.

Test for saponins: The froth test was used to test for saponins and involved adding 3 mL of distilled water to 1 mL of extract and shaking vigorously. The formation of foam indicates a positive test.

Test for phenols: The ferric chloride test was used to test for phenols and involved adding a few drops of ferric (III) chloride (reagent grade-Merck, ≥98%) to the extract. The appearance of a dark green colour indicates the presence of phenols.

Test for glycosides: Killer Killian’s test was used to test for glycosides and involved adding 2 mL of acetic acid containing a drop of ferric (III) chloride to 2 mL of the extract. The formation of a brown-coloured ring indicates the presence of glycosides.

Test for terpenoids: Salkowski’s test was used to test for terpenoids and involved adding 2 mL of chloroform (suitable for HPLC-Merck, ≥99.8%) to 5 mL of the extract, followed by adding 3 mL of concentrated sulphuric acid (ACS reagent-Merck, 95.0–98.0%) to the sides of the test tube. The formation of a reddish-brown colouration interface indicates the presence of terpenoids.

Test for proteins: The biuret test was used to test for proteins and involved adding a few drops of 4% sodium hydroxide and 1% copper sulphate (Reagent Plus-Merck, ≥99%) solution to 2 mL of the extract. The appearance of a violet colour indicates the presence of proteins.

### 3.3. Synthesis of ZnO NPs

**Figure 11:**
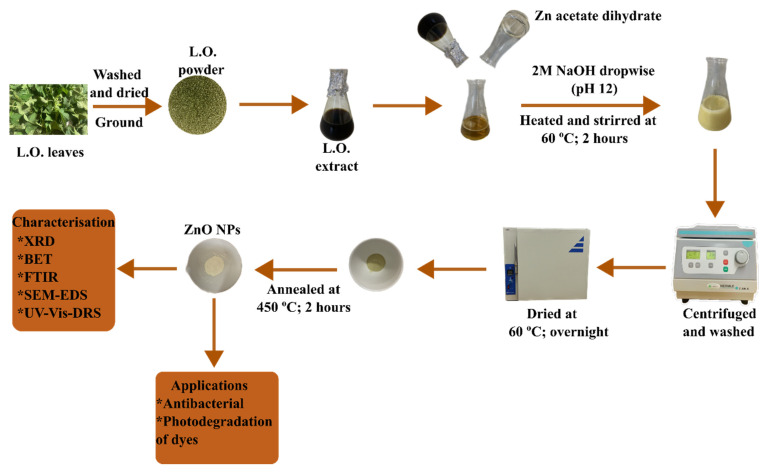
Schematic of the preparation of the L.O-mediated ZnO NPs.

The L.O-mediated ZnO NPs were synthesised by adding 20 mL of L.O. extracts to 100 mL of 0.1 M zinc acetate dihydrate (analytical grade-Merck, 98%), and the mixture was heated to 60 °C. The pH of the mixture was adjusted to 12 by adding sodium hydroxide dropwise and then maintaining the mixture at 60 °C for 2 h. The mixture was then centrifuged, and the obtained precipitate was washed twice with distilled water followed by ethanol. The precipitate was dried in the oven at 60 °C overnight and annealed at 400 °C for 2 h to obtain the L.O-mediated ZnO NPs. Optimisation of this synthesis was carried out by varying the key reaction parameters, including pH (6, 8, 10, and 12), reaction temperature RT, 40, 60, and 80 °C), precursor concentration (0.05, 0.1, 0.2, and 0.4 M), and the volume of the L.O. extracts (20, 40, 80, and 100 mL). Following optimisation, ZnO NPs were synthesised using different zinc salt precursors, including zinc sulphate heptahydrate (ACS reagent-Merck, 99%) (Z001) and zinc acetate dihydrate (Z002), while ZnO NPs synthesised without L.O. extracts were labelled Z003. Figure 11 provides a schematic representation of the synthesis process for the L.O-mediated ZnO NPs.

### 3.4. Characterisation Techniques

The characterisation of the synthesised ZnO NPs was performed using various techniques to assess their optical, structural, and morphological properties. The UV-Vis spectra were obtained between a 300–800 nm wavelength with a 1 nm resolution using a Hach DR6000 UV-Vis spectrophotometer. This was used to confirm the formation of ZnO NPs and to study the optimisation of the plant-mediated synthesis process. DRS spectra were obtained using a HITACHI U-3900 UV-Vis spectrophotometer with a wavelength range of 190 to 900 nm and at a 0.5 spectral bandpass. XRD patterns were obtained using a Rigaku Miniflex 600 (Cukα) operating with an electron energy of 40 keV, a scan range of 10–90˚, and a scan rate of 0.01 min^−1^. FTIR spectra were obtained at RT using a Perkin Elmer FTIR covering a wavelength range of 400–4000 cm^−1^ with a 2 cm^−1^ resolution. The morphology, particle size, particle size distribution, and elemental composition of the ZnO NPs were examined using a SEM equipped with EDS, specifically the SEM-EDS TESCAN VEGA model from JEOL, Peabody, MA, USA, with an accelerating voltage of 2 kV. The surface area and porosity were studied via nitrogen adsorption-desorption BET analysis using a Micromeritics Tristar II 3020 model from Micromeritics, Norcross, GA, USA, at a pretreatment temperature of 150 °C and a run time of 300 min.

### 3.5. Photocatalytic Degradation Studies

The photocatalytic activity of the L.O-mediated ZnO NPs was evaluated by measuring the decomposition of MB in an aqueous solution under UV irradiation. The photocatalysis setup was done following the setup reported by Mugumo et al. [54]. The experiment was conducted by placing 100 mg of L.O-mediated ZnO NPs in 100 mL of 10 ppm of MB solution in a 250 mL beaker. The mixture was placed in a dark room for 30 min and stirred to reach equilibrium. After this initial period, 2 mL of the solution was taken and analysed with a UV-Vis Biochrom WPA Lightwave II spectrophotometer. The remaining solution was exposed to an 18 W UV A light placed 15 cm from the sample and stirred continuously for the duration of the experiment. At 30 min intervals, 2 mL was withdrawn and analysed to monitor the degradation process. The degradation efficiency was calculated as follows:

(4) $$\%\;degradation=\frac{C_{0}-C}{C_{0}}\times 100,$$

where *C*_0_ and *C* are the concentrations of the MB at time *t* = 0 and *t*, respectively.

### 3.6. Antibacterial Activity Studies

The antibacterial activity assessment of the L.O-mediated ZnO NPs against G− *E. coli* and G+ *S. aureus* was conducted following the agar-well diffusion method. The method involved boring wells of about 6 mm deep using a sterile borer into the agar plates that were inoculated with a standardised inoculum of the bacteria. The wells were then filled with the suspension of 5 mg/mL of L.O-mediated ZnO NPs and incubated at 37 °C for 18 h in an incubator. After the incubation period, the antibacterial activity was assessed by measuring the diameters of the clear zones around the wells, which represent the zones of inhibition (ZOI). The sizes of these inhibition zones, recorded in millimetres (mm), indicate the effectiveness of the L.O-mediated ZnO NPs against the bacterial strains [55].

## 4. Conclusions

This study reported, for the first time, the use of an indigenous plant in southern and eastern Africa, L.O., for the green synthesis of ZnO NPs. The XRD, DRS, and EDS results revealed the crystallite sizes of 18.24–21.50 nm, with estimated bandgaps of 3.07–3.18 eV, and confirmed the formation of the ZnO NPs, respectively. The proposed synthesis approach using L.O. extracts resulted in better properties of the synthesised ZnO NPs, as evidenced by the SEM results, which revealed average particle sizes of 56.3 nm for Z001 and 68.8 nm for Z003. The biomolecules such as saponins, tannins, and flavonoids from the L.O. extracts resulted in better particle size control. The FTIR spectra of the L.O-mediated ZnO NPs revealed the presence of functional groups on the surface of the synthesised ZnO NPs that could be attributed to biomolecules such as phenols, tannins, and flavonoids. The better properties of L.O-mediated ZnO NPs than chemical-mediated ZnO NPs, and the presence of biomolecules from the L.O. extracts on the surface of the L.O-mediated ZnO NPs, resulted in their superior photodegradation and antibacterial performances than chemical-mediated ZnO NPs. Z001 exhibited a degradation efficiency of 89.81% against MB dye in 240 min under UV irradiation compared to 56.13% for Z003, whereas the ZOI of Z001 and Z003 were 12 and 7 mm, respectively, against *S. aureus*. Additionally, using different Zn salt precursors to prepare ZnO NPs affected their properties, as evidenced by the differences in the average particle sizes from the SEM and BET surface areas of Z001 (56.3 nm and 20.3 m^2^g, respectively) and Z002 (61.0 nm and 18.0 m^2^g, respectively). This also resulted in their differences in performances in photodegradation applications. Z001 exhibited better photodegradation efficiency compared to Z002 (83.19%), whereas their antibacterial activity was comparable. Optimisation of the photodegradation of MB dye using Z003 under UV irradiation for 180 min revealed a degradation efficiency of 99.89% at the optimum pH, dosage, and initial concentrations of 11, 80 mg and 10 ppm, respectively. Additionally, Z001 showed stability after four degradation cycles and, therefore, L.O-mediated ZnO NPs have the potential to be used as photocatalysts in the degradation of MB dye. These findings underline the potential of the green synthesis of ZnO NPs using extracts of understudied and underutilised plants in southern Africa. This synthesis approach not only provides an environmentally friendly alternative to conventional chemical synthesis, but it also improves the properties of NPs, resulting in better performances in their applications.

## Figures and Tables

**Table 1:** Qualitative analysis of the L.O. extracts.

Phytochemical	Results
Flavonoids	Positive
Alkaloids	Negative
Phenols	Positive
Tannins	Positive
Saponins	Positive
Terpenoids	Positive
Glycosides	Positive
Proteins	Negative

**Table 2:** The optical properties of the synthesised ZnO NPs.

Sample	Absorption Edge (nm)	Bandgap (eV)
Z001	425.68	3.18
Z002	439.48	3.13
Z003	450.89	3.07

**Table 3:** BET results of the synthesised ZnO NPs.

Sample	BET Surface Area (m^2^g)	Pore Volume (cc/g)	Pore Diameter (nm)
Z001	20.3	0.0336	10.6
Z002	18.0	0.0269	8.4
Z003	15.7	0.0223	8.5

**Table 4:** Antibacterial activity of the synthesised ZnO NPs, positive control, and negative control against *S. aureus*.

Sample	ZOI (mm)
Z001	12
Z002	10
Z003	7
Positive control	19
Negative control	Nil

## Data Availability

The original contributions presented in the study are included in the article, further inquiries can be directed to the corresponding author.
